# Assessment of Prescription Completeness and Drug Use Pattern in Tibebe-Ghion Comprehensive Specialized Hospital, Bahir Dar, Ethiopia

**DOI:** 10.1155/2020/8842515

**Published:** 2020-10-19

**Authors:** Zewdu Yilma, Tena Mekonnen, Ebrahim Abdela Siraj, Zegaye Agmassie, Adane Yehualaw, Zenaw Debasu, Chernet Tafere, Misgan Ararsie

**Affiliations:** Department of Pharmacy, College of Medicine and Health Sciences, Bahir Dar University, Bahir Dar, P.O. Box 79, Ethiopia

## Abstract

**Introduction:**

Irrational medicine use is a global problem, and one of its manifestation is inappropriate prescribing that occurs when medicines are not prescribed in accordance with the guideline.

**Objective:**

The aim of the study was to assess prescription completeness and drug use pattern of the hospital using the WHO core drug use indicators.

**Methods:**

1000 prescriptions were collected retrospectively from prescriptions written for 1 year from outpatient pharmacies of the hospital. Exit interview was employed to assess patient care indicators. The health facility indicators were checked by assessing the presence of drug formulary and availability of key medicines at the facility. Data were analyzed using SPSS version 20.

**Results:**

It was found that only name of the patients was filled in all the prescriptions. Other informations were below the standard. The average number of drugs per prescription was 1.65. Percentages of encounter by generic name, with antibiotic and injections, were 85.78%, 41%, and 25%, respectively. The percentage of drugs prescribed from an essential drug list was 98.48%. The mean consultation time and dispensing time were 14.49 and 2.16 minutes, respectively. More than half patients had knowledge on drug dispensed to them (68%). The percentage of drugs actually dispensed was 65%, but none of the drugs dispensed were adequately labelled. A copy of EDL and 84% of the key drugs were available in the hospital.

**Conclusion:**

From the results of our study, it can be concluded that all prescriptions were not complete, and except the average number of drugs prescribed per encounter, the other drug use pattern indicators were out of the WHO recommendation. Therefore, effective intervention program, like training, for promotion of rational drug use practice was recommended.

## 1. Introduction

Medicines are substances or a mixture of substances used for prevention, diagnosis, mitigation, or treatment of disease [1]. Most leading cause of death and disability in developing countries can be prevented, treated, or at least alleviated with cost effective essential medicines. Despite this fact, hundreds of millions of people do not have regular access of these medicines, and many of those who have access are using it irrationally. Irrational medicine use occurs with polypharmacy, with the use of wrong or ineffective medicine, or with under use or incorrect use of effective medicine. These actions negatively affect the quality of drug therapy and medical care, raise health care costs, and may cause adverse reaction, as well as being a primary contributor to the spread of antimicrobial resistance [2].

Medicine use is a complex activity involving the interaction of different bodies like health professionals, the patient (client), and health institutions. All involved in the therapeutic process contribute to irrational use in a variety of ways [3]. Inappropriate prescribing is one of the manifestation of irrational medicine use that occurs when medicines are not prescribed in accordance with the guideline. All the necessary information in the prescription should be completed by the prescribers, since incomplete information could lead to poor treatment outcome and be harmful to the patient. In order to say a given prescription paper is complete, all parameters that are indicated in the prescription paper have to be completed by the prescribers [2, 4].

In order to design different interventional strategies attempting to change medicine use, the scale of the problem should be assessed and quantified. Drug utilization research with the WHO drug use indicators is becoming increasingly necessary to promote rational medicine use and to identify problems related to medicine use especially in developing countries, like Ethiopia, to ensure that the scarce resources are utilized in the best possible manner. Conducting periodic studies of pattern of drug use in various hospital settings or patient populations is essential to identify specific medicine use problems, sensitize practitioners on rational medicine prescription, and to critically analyze the current hospital drug policies and to make recommendations based on various guidelines to improve upon the current drug usage pattern [5, 6].

Studies done in Alexandria (Egypt) [7], India [8], Jordan [9], and selected public hospitals of eastern Ethiopia [10], as an example, showed that most of the WHO drug utilization core indicators were below the optimum value. This indicates that periodic assessment and intervention is necessary in every health settings in order to have and maintain the use of drugs rationally.

The current study was, therefore, designed to identify the major problems in prescription completeness and rational use of drugs in Tibebe-Ghion comprehensive specialized hospital, by using WHO indicators. This investigation plays a major role to prioritize the main intervention areas regarding rational use of medicines. It might provide baseline information for researchers who are interested to conduct further studies to determine factors for drug use pattern in this facility.

## 2. Methods

### 2.1. Study Area and Period

The study was conducted in Tibebe-Ghion comprehensive specialized hospital, a teaching hospital under college of medicine and health sciences of Bahir Dar University located in Bahir Dar, Ethiopia. The Pharmacy Service Directorate is one of the seven directorates found in the hospital. The study was conducted from April to May 2020.

### 2.2. Study Design

Institutional based cross-sectional study design was used to collect the quantitative data from prescription papers dispensed to outpatients between April 1, 2019, and April 1, 2020 (1 year). Prescriptions which contained only drugs and drugs with medical supplies and dispensed to outpatients were included in this study. However, the investigation excluded inpatient prescriptions, prescriptions with only medical supplies, fluids, and/or parenteral nutrition. According to the WHO guide, at least 600 encounters should be included in a cross-sectional survey to describe the current prescribing practices, with a greater number if possible [11]. Therefore, prescribing indicators were assessed retrospectively using 1000 prescriptions selected by randomly among prescriptions filled between April 1, 2019, and April 1, 2020. Patient care indicators were assessed prospectively by conducting exit interview for 100 patients at the outpatient pharmacy of TGCSH between April and May 2020. Health facilities were assessed through observation in order to assess the availability of drug formulary and key medicines at the facility during study period.

Patients included for patent care indicator study were those who attend in outpatient pharmacy and willing to participate. Those who were severely ill, unable to talk, and who were not willing to participate were excluded from this study. All the three groups of indicators were assessed based on the WHO/International Networks for Rational Use of Medicines (INRUD) guidelines [3].

To assess the availability of key medicines in the hospital, 25 medicines were included according to modified Federal Ministry of Health tracer Medicines list [12].

### 2.3. Data Collection and Analysis

Statistical Packages for Social Sciences (SPSS) version 20 was employed for entry and analysis of the quantitative data. In the statistical analysis, frequencies and percentages were obtained. The findings were interpreted according to standard values of WHO prescribing indicators [3].

### 2.4. Ethical Approval

A support letter was obtained from College of Medicine and Health Science before conducting the study. TGCSH Pharmacy Service Directorate gave permission to undertake the study. Consent of each participants of the study was taken before they were interviewed. Individuals participating in the study were informed about the purpose of the study. Study participants with inadequate knowledge were advised on how to take their medicine properly after the interview.

## 3. Results

### 3.1. Prescription Completeness

In order to assess the prescription completeness, patient related, treatment related, prescriber related, and dispenser related information were considered. Principally, each information in the prescription should be filled. In this study, it was found that only full name of the patient was filled in all the prescription seen. The recordings with respect to patient weight, address of the patient, and dosage form of the drug were below 10%. Except dosage form and total drug dose, other parameters with regard to treatment information were above 61%. From professional information, relatively better practice was found in prescribers than dispensers (Table 1).

### 3.2. Prescribing Indicators

One thousand prescriptions were analyzed, and a total of 1650 prescribed drug products were obtained. The average number of drugs per prescription was 1.65. The total number of drugs prescribed by generic name was 1415 (85.78%). Antibiotics were prescribed in 410 (41%) encounters, and injections were prescribed in 250 (25%) prescriptions. 1625 (98.48%) drugs prescribed were from essential drug list (Table 2).

Out of 1650 drugs prescribed, 445 (26.97%) were antibiotics. The three most commonly prescribed antibiotics were ciprofloxacin 83 (18.65%), ceftriaxone 82 (18.43%), and metronidazole 50 (11.24%) (Table 3). Of the total drugs prescribed, 515 (30.28%) were in the form of injections. The first three most commonly prescribed injections were ceftriaxone 82 (17.67%), diclofenac 45 (9.70%), and metronidazole 30 (6.47) (Table 4).

### 3.3. Patient Care Indicators

One hundred patients were considered to analyze patient care indicators and found that an average of 14.49 minutes was taken to consult the patient and 2.16 minutes for dispensing the prescribed drugs to the patient. Out of the total 200 drugs prescribed, only 130 (65%) were actually dispensed with no adequate labeling. Of the patients interviewed, 68 (68%) had knowledge on the correct dose (Table 5).

### 3.4. Facility Specific Indicators

In the hospital pharmacy, the essential drug list is available, and there are 25 drugs which are considered as key drugs. During the study period, only 21 drugs (84%) were available (Table 6).

## 4. Discussion

### 4.1. Prescription Completeness

Prescription order is an important transaction between the physician and the patient. Therefore, it should be written legibly, accurately, and completely in order to minimize errors in the dispensing and administration of medications. In order to say the prescription paper is complete, all parameters that are indicated in the prescription paper have to be completed by the prescribers. These are patient information (patient full name, sex, age, weight, card number), treatment information (medicine full name in generic, strength, dosage form, dose, frequency, duration of treatment), and professionals' information (prescriber's full name, qualification and signature, dispenser's full name, qualification and signature, date of prescribing and dispensing) [5, 13].

It is recommended that any prescriber and dispenser should fill/record the required information (i.e., patient, treatment, and professionals' information) on the prescription paper. But in this study, except the name of the patient, all the assessed prescriptions were incomplete which contained at least one or more unfilled parameters. If we look at the patient information, only 0.4%, 1.9%, and 31% of prescriptions had weight, address, and diagnosis of the patient, respectively. The other patient-related information were above 98%. Out of the treatment information, the least records were found for dosage form type (8.3%) and total drug dose (37.3%). The remaining treatment-related information were found 61% and above. With regard to the professionals' information, though the recordings were below the required (100%), prescribers' information were well recorded relative to the dispensers' information (Table 1). Although there were poor practices that has to be corrected, compared to other studies done elsewhere [5, 13], our study revealed the presence of relatively better practice in the hospital in almost all prescription information parameters.

### 4.2. Prescribing Indicators

Inappropriate use of drugs occurs all over the world and causes harm to people [7]. In our study, WHO/INRUD drug use indicators were used to describe current treatment practices that are helpful for problem identification, detect whether a facility is exceeding or under a set norm of practice [3], and serve as a baseline information for continuous monitoring for ongoing basis in the hospital.

In the current study, the average number of drugs per encounter was 1.65, which is within the recommended limit by the WHO/INURD [11]. Compared to other studies conducted in Ethiopia, like Ayder referral hospital (2.61) [14], Debremarkos Hospital (2.4) [15], five national regional states (Tigray, Amhara, Oromia, SNNPR, Benishangul-Gummuz), and Addis Ababa (1.99) [16], and abroad like Kenya 2.7 [17], Nigeria 3.04 [18], India 3.11 [19], Ghana 4.8 [20], Bahrain 3.3 [21], and United Arab Emirates (UAE) 2.49 [22], the presented study showed better prescribing practice regarding the number of drugs per prescription.

Low generic prescribing practice is observed in our study; out of 1650 drugs, only 1415 (85.78%) drugs were prescribed with their generic name. This value is lower than that of the WHO recommendation (100%) [11]. Higher values were obtained from studies conducted in Hawassa University teaching and referral hospital 98.7% [23], Felege Hiwot referral hospital 97.4% [24], and Ayder referral hospital 93.3% [14]. However, our study revealed relatively better prescribing practice than from studies done in Debremarkos Hospital 77.7% [15], four west Ethiopia public hospitals (Ambo, Gedo, Nekemet, Gimbi) 79.2% [25] and Borumeda hospital 80.02% [26]. Some studies conducted abroad showed much smaller generic prescribing practice than from the current study, for example, in Kenya 45.5% [17], Nigeria 42.7% [18], Pakistan 71.6% [27], Nepal 59.02% [28], Jordan 57.6% [29], and Uzbekistan 38% [30]. But generic use in India 96.88% [19] and UAE 100% [22] was higher.

Overuse of antibiotics is not recommended in any health facility or community, because it develops resistance, which is called antimicrobial resistance [31]. In the current study, antibiotics were prescribed in 410 (41%) of the total prescriptions which is higher to WHO recommendation (20-26.8%) [11]. Higher values were also found in studies conducted at Debremarkos Referral Hospital (71.36%) [15], Hawassa University teaching and referral hospital (58.1%) [23], five national regional states (Tigray, Amhara, Oromia, SNNPR, and Benishangul-Gummuz) and Addis Ababa (58%) [16], and four west Ethiopia public hospitals (Ambo, Gedo, Nekemet, Gimbi) (54.7%) [25]. However, compared to our study, lower values were seen in Felege Hiwot hospital 38% [24], Borumeda hospital 34.57% [26], and Ayder referral hospital 32% [14]. Studies done in Kenya, Ghana, Bahrain, and Pakistan had resulted values of 74%, 60%, 45.8%, and 48.9%, respectively [17, 20, 21, 32]. In the contrary, better results which were within the limit of WHO recommendation were found in studies conducted at an Indian hospital, in health facilities of Jordan and UAE with values of 22.19%, 17.7%, and 9.8%, respectively [19, 22, 29].

Irrational use of injections is the means for transmission of very serious blood-borne infections and leads to morbidity and death. In the current study, injections were prescribed in 250 (25%) of the total prescriptions. This value is close to, but a bit higher, the WHO recommendation (13.4-24.1) [11]. A study done in Debremarkos referral hospital showed that 48.36% prescriptions had injectable [15], 42.2% was found in Mekelle General Hospital [33], and 38.1% was revealed in Hawassa University teaching and referral hospital [23].

An essential medicine list is one of the key tools for applying rational drug use, and use of such a list for any community-based health-care program has a major role in the effectiveness of therapy [8]. In our study, out of the total prescribed drugs, 98.48% of drugs were listed in the hospital's essential drug list. This result is less than from the recommended value by WHO (100%) and other study reports from different health facilities in Ethiopia, like Felege Hiwot referral hospital and Ayder referral hospital which adhered 100% with EML [14, 24] but comparable to the studies done in Debremarkos referral hospital and Hawassa University teaching and referral hospital, whose results were 98.24% and 96.6%, respectively [15, 23].

### 4.3. Patient Care Indicators

The time spend by prescribers and dispensers with each patient sets important limits on the potential quality of diagnosis and treatment. Patients for whom drugs are prescribed should, at a minimum, receive well-labeled medications and should understand how to take each drug.

In our study, the average time that the patient spent with the prescriber was about 15 minutes. This consultation time is better than from other studies done elsewhere, Pakistan (22 min) [27], Egypt (7.1 min) [7], and selected hospitals in eastern Ethiopia (4.61 min) [10], but lower than a study done in Malaysia (18.2 min) [34]. Slower and longer consultation is associated with doctors being more likely to identify psychosocial problems, explore presenting complaints more accurately, prescribe less, and offer more preventative advice [34].

Time spent with the dispenser was found to be 192.6 secs which can be considered very enough compared to other studies done in selected public hospitals in eastern Ethiopia (61.12 sec) [10], Pakistan (38 sec) [27], and Egypt (47.4 sec) [7]. Comparable, even better, result was reported in private tertiary care teaching hospital in India (244 sec) [35].

In order to measure the degree to which the hospital is able to provide the drugs which is prescribed, the dispensers record essential information on the drug packages they dispense and to measure the effectiveness of the information given to patients on the dosage schedule of the drugs they receive; percentage of drugs actually dispensed, adequately labelled, and patients' knowledge of correct dose were studied. The percentage of drugs actually dispensed was 65%. This value is less than from what is ideally recommended (i.e., 100%) and from studies done in Pakistan (90.9%) [27], Egypt (95.9%) [7], and private tertiary care teaching hospital in India (95.5%) [35]. In our study, it was found that no drug was adequately labelled (0%). This was due to inadequate availability of the packaging materials in the studied health facility. Due to this, only oral information had been given to the patients. The percentage of patients' knowledge of correct dose was studied and resulted 68%, which was higher than a study in private tertiary care teaching hospital in India (31%) [35] and comparable to the value found in Pakistan (62.1%) [27] but less than those of results obtained from studies in selected hospitals in eastern Ethiopia (75.7%) [10] and Egypt (94%) [7].

### 4.4. Facility-Specific Indicators

Our study revealed that the hospital had a copy of EDL/formulary which is in agreement with the recommended value (optimal value 100%). The availability of these materials is vital for health professionals for continuous professional improvement and good patient outcomes. The problem was not yet distributed to health professionals. Similar result was obtained in study conducted at Tikur Anbesa Specialized Hospital [5]. On the other hand, the percentage of key drugs in the stock was 84%. This value was better than a finding from selected public hospitals in eastern Ethiopia (66.7%) [10] and Egypt (78.3%) [7] and lower than that of a study in private tertiary care teaching hospital in India (91.6%) [35] and that of the recommended value (100%), but it was in concord with a study in Pakistan (82%) [27]. Limited availability of key drugs might be associated with budgetary constraints, inadequate drug supply system, or poor inventory management of the responsible staff [27].

## 5. Conclusion

From the results of our study, it can be concluded that all prescriptions were not complete. Except the name of the patient, which was recorded in all the prescriptions considered in this study, the other patient related, treatment/drug related, and professionals' related information were below the standard. In addition, most of the WHO core drug use indicators were not met by the hospital. Except the average number of drugs prescribed per encounter, the consultation time, the dispensing time, and availability of copy of EDL, the other indicators were out of the WHO recommendation. Specially, there was no drug which was adequately labeled at all. Therefore, effective intervention program for promotion of rational drug use practice is recommended. The hospital has to take into account this finding and arrange a training program by which each and every medical and pharmacy staff will get an update on rational use of drugs and act accordingly. In addition, there should be a continual assessment on the rational use of drugs in the hospital.

## Figures and Tables

**Table 1:** Prescription completeness assessment at Tibebe-Ghion comprehensive specialized hospital from April 2019 to April 2020 (*n* = 1000).

S/N	Patient information		Treatment information		Professional information			
					Prescribers		Dispensers	
	Parameters	%	Parameters	%	Parameters	%	Parameters	%
1	Full name	100.0	Drug name	99.5	Full name	88.5	Full name	55.4
2	Sex	99.4	Drug strength	67.5	Qualification	49.3	Qualification	11.2
3	Age	98.8	Drug dose	61.3	Date	89.4	Date	40.1
4	Weight	0.4	Total drug dose	37.3	Signature	95.5	Signature	78.7
5	Card #	99.1	Frequency	87.8				
6	Diagnosis	31.0	Duration	75.3				
7	Address of the patient	1.9	Dosage form	8.3				

**Table 2:** Drug prescribing indicators at Tibebe-Ghion comprehensive specialized hospital from April 2019 to April 2020 (*n* = 1000).

Prescribing indicators	Number	Average/percentage	Ideal WHO value [7]
Average number of drugs per encounter	1650	1.65	1.6-1.8
Percentage of drugs prescribed by generic	1415	85.78%	100%
Percentage of encounter with antibiotics	410	41.00%	20.0-26.8%
Percentage of encounter with injections	250	25.00%	13.4-24.1%
Percentage for drugs from essential drug list	1625	98.48%	100%

**Table 3:** Commonly prescribed antibiotics at Tibebe-Ghion comprehensive specialized hospital from April 2019 to April 2020 (*n* = 1000).

Prescribed antibiotics	Frequency of	Percentage
Ciprofloxacin	83	18.65
Ceftriaxone	82	18.43
Metronidazole	50	11.24
Amoxicillin	29	6.52
Azithromycin	29	6.52
Amoxicillin with clavulanic acid	28	6.29
Cephalexin	24	5.39
TTC eye ointment	19	4.27
Cloxacillin	16	3.60
Vancomycin	14	3.15
Ampicillin	9	2.02
Gentamicin	9	2.02
Doxycycline	8	1.80
Cefixime	7	1.57
B. penicillin	6	1.35
Oxytetracycline	6	1.35
Griseofulvin	5	1.12
Cefepime	4	0.90
Chloramphenicol	4	0.90
Hydrogen peroxide	4	0.90
Norfloxacine	3	0.67
Aciclovir	1	0.22
Ceftazidime	1	0.22
Clarithromycin	1	0.22
Clotrimazole	1	0.22
Erythromycin	1	0.22
Nitrofurantoin	1	0.22
Total	445	100

**Table 4:** Commonly prescribed injections at Tibebe-Ghion comprehensive specialized hospital from April 2019 to April 2020 (*n* = 1000).

Prescribed injection	Frequency	Percentage
Ceftriaxone	82	17.67
Diclofenac	45	9.70
Metronidazole	30	6.47
Tramadol	29	6.25
Insulin NPH	23	4.96
Cloxacillin	19	4.09
Lidocaine	18	3.88
Dextrose 40% glucose	16	3.45
Vancomycin	13	2.80
Dexamethasone	12	2.59
Ranitidine	12	2.59
Atropine	11	2.37
Tetanus antitoxin	11	2.37
Ampicillin	9	1.94
Cimetidine	9	1.94
D5%W	9	1.94
Gentamycin	9	1.94
Oxytocin	9	1.94
Ciprofloxacin	8	1.72
Heparin	8	1.72
Metoclopramide	8	1.72
Omeprazole	7	1.51
Regular insulin	7	1.51
Ringer lactate	7	1.51
B. penicillin	6	1.29
Pethidine	6	1.29
Furosemide	5	1.08
Diazepam	4	0.86
Hydrogen peroxide	4	0.86
Mannitol	4	0.86
Haloperidol	3	0.65
Hydrocortisone	3	0.65
Hyoscine	3	0.65
KCl	3	0.65
Adrenaline	2	0.43
Cefepime	2	0.43
Dextrose in normal saline	2	0.43
Artesunate	1	0.22
Bupivacaine	1	0.22
Ceftazidime	1	0.22
Magnesium sulphate	1	0.22
Potassium chloride	1	0.22
Vitamin B complex	1	0.22
Total	464	100

**Table 5:** Patient care indicators at Tibebe-Ghion comprehensive specialized hospital from April 2019 to April 2020 (*n* = 100).

Patient care indicators	Values
% consultation time	14.49 min
% dispensing time	2.16 min
% drugs actually dispensed	65%
% drugs adequately labeled	0%
Patients' knowledge of correct dose	68%

**Table 6:** Facility specific indicators at Tibebe-Ghion comprehensive specialized hospital from April 2019 to April 2020.

Facility specific indicators	Availability in the hospital	Distributed to the staff (Y/N)
Availability of copy of EDL or formulary	Available	No
% availability of key drugs in the stock	84%	No

Y/N = Yes/No.

## Data Availability

The authors confirm that data used to support the findings of this study are available from the corresponding author upon request.
